# Supplementary material for: A lithium-ion batteries SOH estimation method based on extracting new features during the constant voltage charging stage and improving BPNN
Source: PLoS One. 2025 May 23;20(5):e0324868. doi: 10.1371/journal.pone.0324868 (PMC12101645; doi:10.1371/journal.pone.0324868)
Supplement: Attachment — Submitted filename: Response to Reviewers.docx [file pone.0324868.s003.docx]

Dear Editors and Reviewers:

We are sincerely grateful for your time and effort in reviewing our manuscript. Your insightful comments have been instrumental in guiding us to enhance the quality and clarity of our work. We have carefully considered each of the reviewers' comments and have made every effort to incorporate the suggested changes into the revised manuscript. These revisions have refined our presentation and improved readability without altering the core content or framework. We have addressed each comment individually and provided detailed explanations of the changes we have made. We hope that these revisions meet with your approval and demonstrate our commitment to producing a work that meets the high standards of the journal.

Once again, we thank you for your dedication to maintaining the integrity and excellence of academic publishing. We are eager to hear your further thoughts and are confident that the revised manuscript addresses all concerns raised during the review process.

**Reviewer #1**

The manuscript presents a method for estimating the SOH of lithium-ion batteries by extracting health features (HF) during the constant voltage (CV) charging stage and optimizing a Backpropagation Neural Network. While the approach shows potential, several issues in the paper need to be addressed to improve clarity and rigor. I recommend major revisions for the manuscript to address these issues.

**Response:**

We sincerely appreciate the time and expertise you have dedicated to evaluating our manuscript. Your insightful comments have greatly contributed to strengthening the academic rigor and clarity of this work. We have carefully read each of your concerns with the utmost attention and made substantial revisions to enhance the clarity, rigor, and scientific contribution of the paper. The detailed responses to your comments are given as follows.

**1.Comment:**

In the abstract, the statement "Existing methods for estimating the state of health (SOH) of lithium-ion batteries primarily extract health features (HF) during the constant current (CC) charging stage." is inaccurate. Many studies also extract features during the CV stage. This needs to be revised for accuracy.

**1.Reply:**

Thank you for your careful review of the research background of this article. You pointed out that many studies are very accurate in extracting features during the CV stage. Our correction to this issue is as follows:

**Original：**

Existing methods for estimating the state of health (SOH) of lithium-ion batteries primarily extract health features (HF) during the constant current (CC) charging stage. However, this approach is susceptible to the randomness of the initial charging stage.

**Revised:**

Existing state of health (SOH) estimation methods for lithium-ion batteries predominantly extract health features (HF) from constant current (CC) and constant voltage (CV) charging phases. Nevertheless, CC charging phase feature extraction is susceptible to the randomness of the initial charging stage.

**2.Comment:**

Why emphasize "Backpropagation Neural Network" (BPNN) instead of just "Neural Network" (NN)? BPNN is a type of NN, so the distinction is unnecessary unless there’s a specific reason for using this term.

**2.Reply:**

Thank you for your professional correction on the standardization of methodological terminology in this article. We understand your concerns and in our paper, we use the term 'BPNN' mainly based on the following aspects：

（1） BPNN is the main method used in our paper for predicting the SOH of batteries, and it is also one of the core technologies we study. We have made in-depth improvements and optimizations to BPNN, especially by combining COA to optimize its weights and thresholds, in order to improve the performance of the model. This improvement is one of the important innovations of the paper, so we emphasize that BPNN is intended to highlight our improvements and applications to this specific neural network model, rather than discussing neural networks in general.
（2） Although BPNN is a type of NN, it has its unique structure and training approach. BPNN is a multi-layer feedforward neural network trained using backpropagation algorithm, capable of handling complex nonlinear problems. In the specific task of battery SOH prediction, we utilize these characteristics of BPNN to establish a mapping relationship between health features and SOH. Emphasizing BPNN can enable readers to have a more accurate understanding of the methods and technical details we use.

We emphasize that 'BPNN' is to accurately convey the core methods used in our research, highlight our innovative points, and avoid possible ambiguity. This is not to say that we deliberately distinguish between BPNN and NN, but rather a reasonable choice based on the content and research focus of the paper. Thank you again for your concern and guidance on the paper. Your feedback has played an important role in improving the quality of our paper.

**3.Comment:**

Contribution 1: "The current curve of the CV charging stage is transformed into a dQ/dI curve, from which two HFs are extracted." This is not a unique contribution, as many papers have done this already. Please revise this point.

**3.Reply:**

Thank you for your rigorous evaluation of the innovative dimensions of this research. We have revised the statement of Contribution 1 as follows, with the aim of accurately stating the specific contribution of the article.

**Original：**

（1） The current curve of the CV charging stage is transformed into a dQ/dI curve, from which two HFs are extracted；

**Revised:**

（1） This study pioneers a novel method for lithium-ion battery health feature extraction within the 200mA threshold during the terminal phase of CV charging；

**4.Comment:**

Ensure that the symbols in equations and the text are consistent in their font style.

**4.Reply:**

Thank you for pointing out this crucial detail. We have conducted a comprehensive review of all formulas and mathematical symbols in the text description, and have now adopted the following format:

(1) Font style: Times New Roma;

(2) Character size: 12 pounds;

(3) Line spacing: Double line spacing.

**5.Comment:**

Regarding the first extracted feature, "sum of dQ/dI data points (SQI)," this is dependent on the sampling frequency and the initial current value during the CV charging stage. Therefore, this feature is not a universal one. Please discuss and clarify this.

**5.Reply:**

Thank you for your attention to the feature extraction section. The concern you raised about the possible dependence of "Sum of dQ/dI data points (SQI)" on sampling frequency and initial current values is reasonable, but we have systematically analyzed and verified its universality from the following perspectives:

（1） In the "Battery datasets" section of this article, the sampling conditions for all test data are clearly defined: the NCA and NCM datasets use constant voltage charging stage data with a 10 second sampling rate, which are sourced from (reference [32]). This rigorous experimental design ensures the consistency of the sampling frequency for SQI calculations and eliminates feature bias caused by differences in sampling rates across datasets. If applied to other scenarios in the future, further universality can be improved through normalization processing (such as standardizing dQ/dI by time integration).
（2） Although SQI seems to rely on the initial current value (*I_si_*) in mathematical definition, there are mechanisms in the actual battery aging process that make it stable：

- The morphological changes of the dQ/dI curve are mainly caused by the internal resistance and polarization effect of the battery (reference [29]), rather than simply determined by the initial current. As the number of cycles increases, the internal resistance of the battery increases, leading to an accelerated decay rate of dQ/dI (as shown in Figure 1);
- By comparing the experimental data at different temperatures (35 ℃ vs 45 ℃) and different rates (0.5C vs 0.25C) (Table 1), it was found that SQI showed a strong linear correlation with capacity degradation (Pearson coefficient>0.94), indicating that its feature extraction ability is not affected by the absolute value of the initial current, but is closely related to the internal state of the battery.

**6.Comment:**

The paper seems to use data from the entire CV charging stage. Why is there constant emphasis on "the end of the constant voltage charging stage"? How is the "end of the constant voltage charging stage" defined?

**6.Reply:**

Thank you for your attention to the logic of data selection. We use data from the entire CV charging stage in this article for visualization and theoretical analysis, revealing the complete evolution mechanism of the dQ/dI curve. But we emphasize 'the end of the constant voltage charging stage' because the health features in this article are only extracted at the moment when CV charging is about to end, in order to meet the real-time requirements and data storage limitations of BMS.

**7.Comment:**

In Equation (4), based on the simplification, shouldn’t IQIC just equal dQ?

**7.Reply:**

Thank you for your attention to the details of the paper. The simplification issue you pointed out is very important, which reflects the rigor of the paper. After careful analysis, we acknowledge that there is a lack of rigor in the wording here. The following is a detailed explanation and correction of this issue:

（1） According to the fundamental theorem of calculus：$\int_{a}^{\text{b}} f'\left(x\right) dx=f\left(b\right)-f\left(a\right)$，therefore $IQIC_{i}=Q\left(I_{ei}\right)-Q\left(I_{si}\right)$，so *IQIC_i_* should be equal to *∆Q*。
（2） We have revised all the parts of the paper that involve this expression to enhance readability and rigor. Thank you again for your valuable feedback!

**8.Comment:**

Why were batteries #1, #2, #9, #10 from NCM and #1, #2 from NCA chosen for testing instead of all available batteries? Was this selection based on the results?

**8.Reply:**

Thank you for your attention to the experimental design. The reason why we choose specific batteries for testing is as follows, rather than preference based on experimental results:

- The dataset provided in reference [32] contains multiple battery labels, but their parameters (such as temperature and charging rate) differ. We only select batteries that meet the following conditions.In the NCM dataset, we selected batteries (# 1, # 2, # 9, # 10) with different temperature conditions (35 ℃ and 45 ℃) to verify the robustness of the method under temperature changes. Similarly, in the NCA dataset, batteries (# 1, # 2) with different charging rates (0.25C) were selected to test the model's adaptability to changes in charging rates. This design ensures that the experiment covers the key variables of temperature and charging rate, thus comprehensively evaluating the generalization ability of the method.
- During the experimental phase, we found that some battery labels in the dataset had missing data. For example, the battery (CY25-1/1 # 2) only had a total of 37 cycles, and the capacity data for the 26th cycle was missing; The rated capacity data for the 51st, 102nd, 153rd, 204th, 255th, and 306th cycles of battery (CY25-0.5/1 # 9) is missing. There are many similar results, which may be due to experimental errors or equipment abnormalities during dataset collection. In order to ensure the authenticity of the original data and avoid misleading conclusions caused by interpolation of the original dataset, we selected batteries with relatively complete original data for testing.

**9.Comment:**

"However, when using BPNN, the initial parameters of the network are set randomly, which may cause the gradient descent algorithm to become trapped in local minima rather than global minima, thereby affecting the network's performance." Is this really an issue? Initialization is not generally a problem in NN training.

**9.Reply:**

Thank you for your attention to the BPNN initialization issue. Although the importance of initialization in modern neural network training may vary depending on the complexity of the model, initialization issues still need to be taken seriously in this study, mainly for the following reasons:

（1） Indeed, the initial parameters of BPNN are randomly set, and this randomness may cause the gradient descent algorithm to get stuck in local minima during the optimization process, rather than finding the global minimum. This situation is particularly common in complex nonlinear problems, especially when the data has high-dimensional features or the network structure is complex. The existence of local minima can affect the performance of the model, leading to inaccurate or unstable prediction results. To ensure the rigor of the content, we have cited relevant literature on this sentence: “However, when using BPNN, the initial parameters of the network are set randomly, which may cause the gradient descent algorithm to become trapped in local minima rather than global minima, thereby affecting the network's performance[15].”
（2） In many cases, using standard initialization methods and optimization algorithms, BPNN can achieve good training results, especially in situations with large data volumes and reasonable feature distributions. However, in some specific cases, optimizing initialization is still necessary. For example, when the amount of data is limited, the features have complex nonlinear relationships, or the network structure is very sensitive to initial parameters, random initialization may lead to instability and performance fluctuations in model training. In our research, we found that optimizing the initial weights and thresholds of BPNN can significantly improve the predictive performance and stability of the model for battery SOH prediction. This is also the reason why we chose to use COA to optimize the initialization parameters of BPNN.

**10.Comment:**

"BPNN is a supervised learning algorithm used to train multi-layer feedforward neural networks." Is BPNN an algorithm or a model? This needs clarification.

**10.Reply:**

Thank you very much for pointing out this important issue of terminology confusion. After careful examination, BPNN is a model rather than an algorithm. We acknowledge that there were some unclear omissions in the initial draft of the paper and sincerely apologize to you. We have revised the original sentence:

**Original：**

BPNN is a supervised learning algorithm used to train multi-layer feedforward neural networks.

**Revised:**

BPNN is a shallow feedforward neural network model based on supervised learning.

**11.Comment:**

As far as I know, regardless of how NN values are initialized, optimization algorithms like SGD or Adam can ensure the model converges well. How do we determine whether the model's optimal point is due to SGD or the COA-initialized values? Since COA requires further optimization through SGD, could you provide evidence to show that the model cannot converge to an optimal point if initialized randomly, and that COA initialization makes a significant difference?

**11.Reply:**

Thank you for reviewing our paper and providing valuable feedback. We understand your concern about how to determine whether the optimal solution of a model is caused by SGD or COA initialization values, and whether evidence can be provided to prove that randomly initialized models cannot converge to the optimal solution, while COA initialization has significant differences. We provide the following explanation for this:

（1） Although optimization algorithms such as SGD or Adam can converge the model in most cases, the initial parameter settings still have a significant impact on the convergence speed and final performance of the model. Different initialization methods may cause the model to converge to different local or global minima during the training process. Especially in complex nonlinear problems, such as battery SOH prediction, the selection of initial parameters may affect the model's ability to capture complex patterns.
（2） Due to the random initialization of weights and thresholds in the BPNN model, each run yields different results, which can lead to unstable results. This is why we use COA to optimize the initial parameters. However, we need to clarify that it does not mean that without optimizing BPNN, it cannot achieve the optimal solution. Instead, through optimization, the parameters generated each time it runs can be relatively stable, because this is the result found after multiple iterations of convergence. Taking CY45-0.5/1 # 10 battery as an example, the fitness curve of COA iterative optimization process is shown in the figure:

The "fitness value" is calculated based on formula (5) in the original text. It can be found that without iterative optimization, the error of the initial parameter value output in the first generation is not the lowest. However, after multiple iterations of convergence, it can ensure that a relatively good set of initial parameter values can be found during each run.

**12.Comment:**

Please avoid using words to represent variables in formulas.

**12.Reply:**

We sincerely appreciate your valuable feedback on the standardization of the formula. After verification, it was found that there were indeed some formulas in the initial draft that used natural language (such as "*Iguana*" and "*local*") instead of mathematical symbols. This is due to insufficient emphasis on symbol unity in the early stages of writing. The full text formula has been comprehensively revised according to academic standards, and the specific modifications are as follows:

(1) Use " *𝜇* " instead of "*Iguana*" in the formula uniformly;
(2) Replace the word '*local*' in the formula with '*φ*' uniformly.

**13.Comment:**

In Equations (14)-(16), please unify the symbols used with those in Equation (5) as they represent the same concepts.

**13.Reply:**

Thank you for your attention to symbol consistency. We have carefully checked the issue of symbol usage between formulas (5) and (14) - (16), and now modify formulas (14) - (16) to match the symbols in formula (5):

**Original：**

$$MAE=\frac{1}{m}\sum_{i=1}^{m} |(\hat{Y}(i)-Y(i))|$$

$$RMSE=\sqrt{\frac{1}{m}\sum_{i=1}^{m} (\hat{Y}(i)-Y(i))^{2}}$$

$$R^{2}=1-\frac{\sum_{i=1}^{m} (\hat{Y}(i)-Y(i))^{2}}{\sum_{i=1}^{m} (Y(i)-\bar{Y}(i))^{2}}$$

**Revised:**

$$MAE=\frac{1}{N}\sum_{k=1}^{N} |(Y_{k}-T_{k})|$$

$$RMSE=\sqrt{\frac{1}{N}\sum_{k=1}^{N} (Y_{k}-T_{k})^{2}}$$

$$R^{2}=1-\frac{\sum_{k=1}^{N} (Y_{k}-T_{k})^{2}}{\sum_{k=1}^{N} (T_{k}-\bar{T}_{k})^{2}}$$

where $\bar{T}_{k}$ represent the mean of expected output, and *k* represents the cycle number.

**14.Comment:**

"The first 70% of the data is selected as the training set, while the remaining 30% is used as the testing set." This validation method is not reasonable. How do you label the first 70% of the data? If the labels can be directly collected or computed, why then use BPNN to predict the remaining 30%? Training on one battery and testing on another (e.g., train on Battery A, test on Battery B) would be a more reasonable setup and closer to real-world applications.

**14.Reply:**

Thank you very much for reviewing our paper and providing valuable feedback. We have carefully considered and analyzed the issues you raised regarding data partitioning and model validation methods. Below is our detailed response:

Firstly, regarding the issue of data partitioning. In this study, we chose to use the first 70% of each battery data as the training set and the last 30% as the testing set, mainly based on the following considerations:

（1） The charging and discharging data of a battery is a continuous process with time series characteristics. Dividing the data of the same battery into training and testing sets in chronological order can ensure the continuity and integrity of the data, enabling the model to learn the characteristics of the battery at different aging stages during the training process and predict the subsequent aging trend during the testing stage；
（2） In practical applications, we usually hope to use existing historical data to train models and then predict future battery states. This partitioning method can simulate the data distribution and prediction scenarios in practical applications to a certain extent, that is, using past operational data to predict future performance.
（3） Due to the relatively limited amount of data per battery, using a method of separating training and testing batteries may result in insufficient training data for each battery, thereby affecting the training effectiveness and generalization ability of the model. Dividing the data of the same battery can to some extent ensure that the model can obtain sufficient training samples. There are also methods in existing research that use this method to partition datasets, such as in references [10] and [15]:

10.Qian C, He N, He L, Li H, Cheng F. State of health estimation of lithium-ion battery using energy accumulation-based feature extraction and improved relevance vector regression. Journal of Energy Storage. 2023;68: 107754. doi:10.1016/j.est.2023.107754

15.	Ma Y, Yao M, Liu H, Tang Z. State of Health estimation and Remaining Useful Life prediction for lithium-ion batteries by Improved Particle Swarm Optimization-Back Propagation Neural Network. Journal of Energy Storage. 2022;52: 104750. doi:10.1016/j.est.2022.104750

However, we also realize that the method you proposed may be more reasonable in certain situations, especially when evaluating the model's adaptability to individual differences in different batteries. In future research, we plan to further explore and attempt this cross validation method to more comprehensively evaluate the performance and generalization ability of the model.

As for the labeling issue you mentioned, we do need further clarification. In this study, the label refers to the actual State of Health (SOH) of the battery, which was measured experimentally. We did not directly use these labels to train the model, but instead used the extracted health features as inputs to estimate the battery's State of Health (SOH) through model learning and prediction. The role of BPNN here is to use the relationship between known health features and corresponding SOH labels to establish a mapping model, which can predict SOH for new and unknown battery data. Therefore, even if we can directly measure or calculate partial SOH labels, we still need to use BPNN to learn and capture the complex nonlinear relationship between health features and SOH, in order to accurately predict unknown battery states.

Thank you again for your attention and valuable feedback on our paper. Your suggestions have important guiding significance for us to further improve our research work. We will continue to strive to improve our research methods, enhance the quality and academic value of our papers.

**15.Comment:**

In Table 2, how were the hyperparameters for the three NN models chosen? What were their specific values? Also, what does "SSA" mean in the “SSA-BPNN” method?

**15.Reply:**

Thank you for reviewing our paper and providing valuable feedback. In response to your question about the hyperparameter selection methods and specific values for the three neural network models in Table 2, as well as the meaning of "SSA" in the "SSA-BPNN" method, we have provided explanations and clarifications. The following is our response:

（1） BPNN：

Number of neurons in the hidden layer: Through multiple experiments and empirical references, we selected 7 neurons as the hidden layer neurons. This is because fewer neurons may lead to underfitting of the model, while too many neurons can increase the complexity and computational complexity of the model, which can easily lead to overfitting. Seven neurons showed good balance in our experiment and were able to fit the nonlinear relationship between battery SOH and health characteristics well;

Learning rate: We adopted a learning rate of 0.01. This learning rate has shown good convergence speed and stability in preliminary experiments. A high learning rate may lead to crossing the optimal solution during gradient descent, while a low learning rate can make the training process too slow.

Maximum iteration count: 3000, which is determined by observing the trend of the loss function.

（2） SSA-BPNN：The parameters of BPNN are unchanged and consistent with the above, because SSA is mainly used to optimize the initial weights and thresholds of BPNN, while the structure and learning rules of BPNN remain consistent to a certain extent, in order to fairly compare the effects of different optimization methods. The SSA related hyperparameters are as follows：

Population size: Set to 50. A population size that is too small may not be able to fully search the solution space, while a population size that is too large can increase the computational burden. A population size of 50 can find a better solution in a reasonable time in our experiment;

Maximum iteration count: set to 300. The number of iterations was determined through multiple experiments, and after 300 iterations, the algorithm was able to find a relatively stable optimal weight and threshold initialization scheme;

（3） COA-BPNN：The parameters of BPNN are the same as above. The hyperparameters of COA are as follows:

Population size: Set to 50. A larger population size helps to search the solution space more comprehensively, and a population size of 50 can better balance computing resources and search efficiency in our experimental environment;

Maximum iteration count: set to 300. This number of iterations is to be consistent with SSA, in order to fairly compare their convergence speed and demonstrate their advantages.

In the SSA-BPNN method, SSA stands for Sparrow Search Algorithm. The sparrow search algorithm is a swarm intelligence optimization algorithm inspired by sparrow foraging and vigilance behavior. We use abbreviations here because we mentioned it in the introduction:“Guo et al. [16] proposed a method for obtaining the weights and thresholds of BPNN using the Sparrow Search Algorithm (SSA).”

**16.Comment:**

The Conclusion section lacks a discussion of the study's limitations and future research directions. These should be added to provide a more balanced perspective.

**16.Reply:**

Thank you for reviewing our paper and providing valuable feedback. We have carefully considered and supplemented the issue of the lack of discussion on research limitations and future research directions in the conclusion section you raised. The following is a detailed supplement, which will be placed at the end of the conclusion:

**Augment:**

Although the COA-BPNN method demonstrated promising performance in experimental datasets, its generalization capability still requires further validation across more diverse datasets and application scenarios. Moreover, the SOH of batteries is a complex multidimensional issue, and there may be other important features related to battery aging that have not been extracted. In the future, we will further research and develop more effective feature extraction techniques to uncover more key information related to health status in battery data.

**Reviewer #2**

**Response:**

First of all, please allow us to express our sincere thanks to you. Your opinion is very valuable to us, it not only provides a new perspective for our research, but also points the way for us to further improve the paper. We know that you have taken time out of your busy schedule to review our manuscripts and give detailed feedback, which shows your concern and support for our research work. Thank you again for your hard work and careful guidance. We have carefully revised and improved the paper based on your feedback, hoping to make the research work more rigorous, substantial, and persuasive.

**1.Comment:**

The main challenges faced by the paper are not reflected in the abstract;

**1.Reply:**

Thank you for reviewing our paper and providing valuable feedback. The issue you pointed out in the abstract that does not reflect the main challenges of the paper is very important, and we fully agree with and greatly appreciate your pointing out this deficiency. In the revised abstract, we have added a description of the main challenges:

**Augment:**

The complexity and noise interference of battery data make it difficult to accurately extract health features, and it is necessary to develop effective methods to process the data and extract representative features.

**2.Comment:**

PINN is a hot topic of research, but the authors do not give any research progress from relevant scholars.

**2.Reply:**

Thank you for reviewing our paper and providing valuable feedback. The issue you pointed out about the research progress of Physics Informed Neural Networks (PINN) is very important, and we fully agree with and greatly appreciate your pointing out this deficiency. In our research, the main focus is on data-driven methods for estimating the SOH of lithium-ion batteries, particularly utilizing the characteristics of the constant voltage charging stage and improved BPNN. However, we did not mention PINN as an important research direction in the paper, which may lead readers to have a less comprehensive understanding of the research progress in the relevant field. In the revised paper, we will add a discussion on the research progress of PINN in the introduction section and cite relevant literature。

**Augment:**

In the recent rise of Physical Information Neural Network (PINN) research, scholars have devoted themselves to combining physical laws with neural networks, providing new ideas and methods for battery health state estimation. PINN incorporates physical equations as soft constraints into the loss function of neural networks, allowing the network to not only learn statistical laws of data during training, but also follow physical laws, thereby improving the accuracy and generalization ability of the model. Relevant scholars have achieved significant research results in this field[18].

18.	Ghosh S, Ogueda-Oliva A, Ghosh A, Banerjee M, Seshaiyer P. Understanding the implications of under-reporting, vaccine efficiency and social behavior on the post-pandemic spread using physics informed neural networks: A case study of China. Lal R, editor. PLoS ONE. 2023;18: e0290368. doi:10.1371/journal.pone.0290368

**3.Comment:**

What are the differences and advantages of COA and other methods, the author does not give in 3.3;

**3.Reply:**

Thank you for reviewing our paper and providing valuable feedback. The issue you pointed out about the lack of detailed explanation of the differences and advantages between COA and other methods is very important, and we fully agree with and greatly appreciate your pointing out this deficiency. We have added a comparison between COA and other optimization algorithms such as SSA:

**Augment:**

For example, SSA may require more iterations to converge, especially when optimizing high-dimensional complex problems, which may affect its convergence speed[16]. Due to its unique search strategy and adaptive parameter adjustment mechanism, COA usually has a faster convergence speed in the optimization process. It can find high-quality solutions in a short number of iterations, which is particularly important for BPNN parameter optimization problems that require a large amount of computation and iteration.

**4.Comment:**

How does the author solve the problem of insufficient dataset, only two features are applied to predict SOH, and the generalization ability is not guaranteed to be applied in practice?

**4.Reply:**

Thank you for reviewing our paper and providing valuable feedback. We understand your concerns about the insufficient dataset, the use of only two features to predict SOH, and the lack of generalization ability, and we provide the following explanations:

（1） The datasets we use, although relatively small, are publicly available and widely recognized, with a certain degree of representativeness and reliability. They cover battery data under different environmental temperatures and charging rates, which can to some extent reflect the performance changes of the battery under different operating conditions. In practical research, data acquisition is often limited by experimental conditions and costs. Therefore, we fully utilize existing data resources and ensure the credibility of research results through rigorous data preprocessing and analysis methods.
（2） The two features we selected are based on in-depth analysis and theoretical derivation of the current curve at the end of the constant voltage charging stage of the battery, and they have a significant correlation with the aging state of the battery. By extracting these two features, we can capture key information about battery aging to a certain extent. In practical applications, feature selection requires comprehensive consideration of data availability, computational complexity, and the impact on model performance. In this study, we focused on exploring the application of these two features in SOH prediction, in order to provide an effective method for rapid assessment of battery health status. We recognize that adding more features may further improve the accuracy and generalization ability of the model. Therefore, in future research, we plan to explore more feature extraction methods to enrich the feature set.
（3） The experimental results show that our model exhibits good predictive performance under different temperature battery and charging rate conditions, which to some extent verifies the generalization ability of the model. We recognize that in practical applications, the types, operating conditions, and aging characteristics of batteries may become more diverse. Therefore, we plan to continuously collect more battery data in future practical applications, train and update the model continuously, in order to improve its generalization ability and reliability in practical applications.

**5.Comment:**

The authors use BPNN as the main method to predict SOH, what is the difference between this and the existing LSTM and TCN?

**5.Reply:**

Thank you for reviewing our paper and providing valuable feedback. In our research, we chose BPNN as the main method to predict SOH, mainly based on the following considerations:

（1） BPNN: The structure is relatively simple, consisting of an input layer, a hidden layer, and an output layer. This simple structure makes BPNN relatively easy to implement and understand, and can achieve good results even in limited data. For the battery SOH prediction problem, we focus on the mapping relationship from extracted health features to SOH, and BPNN can learn this nonlinear mapping through appropriate training. It is suitable for situations where the data volume is relatively small and the feature engineering is relatively clear. In our study, we extracted two representative health features from the end of the constant voltage charging stage of the battery, which showed a significant correlation with the battery's State of Health (SOH). For this feature-based prediction task, BPNN can fully utilize these features for effective learning and prediction.
（2） LSTM：It is a special type of recurrent neural network (RNN) with memory units and gating mechanisms, which can effectively handle long-distance dependencies in sequential data. It performs well in processing data with obvious time series characteristics, such as sequence prediction tasks in natural language processing, speech recognition, and other fields. It is suitable for processing data with complex time series structures, such as battery charging and discharging curves. If the focus of the research is to directly learn the SOH variation pattern of the battery from the original time-series data (such as the time series of voltage, current, etc.), LSTM may play a greater advantage.
（3） TCN：Based on convolutional neural networks (CNNs), techniques such as causal convolution and dilated convolution can be used to capture long-term dependencies in sequence data while maintaining parallel computing capabilities. TCN has high efficiency and performance in processing sequential data, and is suitable for scenarios that require high real-time performance. It is suitable for real-time processing and prediction of sequence data, especially in scenarios that require fast response and efficient computation. If real-time prediction of battery SOH needs to be implemented in embedded systems or online monitoring systems, TCN may be a better choice.

Sincerely,

Mingyang Li
